# Targeting rare tumors: new focus for clinical research in China

**DOI:** 10.15252/emmm.202216415

**Published:** 2022-11-28

**Authors:** Shuhang Wang, Yale Jiang, Huilei Miao, Yuan Fang, Ning Jiang, Yue Yu, Peiwen Ma, Qiyu Tang, Dandan Cui, Hong Fang, Huiyao Huang, Qi Fan, Chao Sun, Anqi Yu, Shuangman Miao, Jingting Du, Jingxiao Zhu, Yuning Wang, Ning Li

**Affiliations:** ^1^ Clinical Trial Center, National Cancer Center/National Clinical Research Center for Cancer/Cancer Hospital Chinese Academy of Medical Sciences and Peking Union Medical College Beijing China

**Keywords:** Cancer

## Abstract

Rare tumor has a huge unmet medical need without standard regimens, calling for novel therapeutic interventions. The National Cancer Center of China identified a threshold of incidence for rare tumor as 2.5/100,000, based on the characteristics of Chinese population. Molecular profiles for rare tumor patients in China further provided prospects for precise and individualized targeted treatment. An ongoing phase II clinical trial, the PLATFORM study, is the first trial tailored for rare solid tumors in China, featured by molecule‐guided therapeutics. With the promulgation of supportive policies to encourage the development of innovative drugs for rare tumors in China, opportunities will be provided for these patients and the gap will be filled in the treatment of rare tumors.

## Graphical abstract

In this timely Commentary, Prof. N. Li and colleagues discuss the challenges related to rare tumors definition, clinical trials, and therapies, with a focus on China.

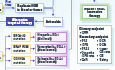

## Definition of ‘rare tumors’ varies between different global regions and China

**Figure 1:**
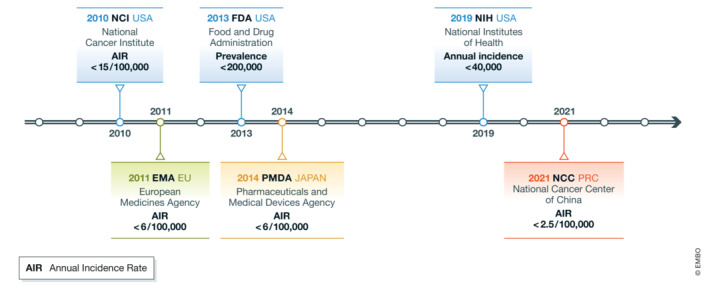
Definition of rare tumor or disease across different countries and regions

During the last decade, a significant improvement in the pathogenetic understanding and treatment of commonly diagnosed cancers has been achieved. The focus is now turning to rare tumors which are still poorly understood. Rare tumors can originate from tissues rarely involved in cancer, such as eyes and testis (type I). They can also originate from tissues where tumor commonly happens but with rare pathology types (type II), such as endometrial stromal sarcoma. Although these cancers are labeled as rare, they still collectively comprise around a quarter of all cancers, yet treatment options are extremely limited owing to the rarity of the patient population of the individual cancers. Currently, there is no global standardized definition for rare tumors. Rare tumors are defined as tumors with an annual incidence rate (AIR) lower than 15/100,000 in the United States (US) by the Food and Drug Administration (FDA), and an incidence lower than 6/100,000 per year in Europe by the European Society for Medical Oncology (ESMO) (Greenlee *et al*, 2010; Gatta *et al*, 2011). In China, there was no definition for rare tumors before 2021, when a research team from the National Cancer Center defined the cutoff as 2.5/100,000 for AIR, based on the prevalence of tumors with inadequate clinical guidelines and on characteristics of Chinese population (Wang *et al*, 2020) (Fig 1).

There are significant inter‐regional differences in the prevalence of various cancers. For example, esophageal cancer, hepatocellular carcinoma, and nasopharyngeal cancers are not uncommon in China with incidence of 18.3, 28.1, and 3.8 per 10,000, respectively, and these cancers are thus absent from the rare tumor list (Zheng *et al*, 2022). By contrast, skin tumors, especially basal cell carcinoma, are otherwise common in western countries and less common in China.

Less than 10% of rare tumor subtypes have explicit guidance in NCCN guidelines with recommended targeted or immune therapies. Given that the disease control rate and objective response rate of targeted therapy outperform standard treatment in rare tumors with targetable mutations, there is a clear need for accurate methods to assess the safety and efficacy of novel clinical interventions against these rare malignancies.

## Potential therapeutic targets for rare tumors in China

Molecular profiling of solid rare tumors in China has been systemically explored with regard to specific mutations or specific tumor types. We have recently compared the incidence of therapeutic targets in rare tumors between the western cohort and our in‐house Chinese cohort (Wang *et al*, 2020). We found that the prevalence of targetable genomic alterations in the Chinese population was significantly higher than in the general population (53.43% vs. 20.40%, respectively), with EGFR ranking top (prevalence of 7.7%). These results indicate an enrichment of potential therapeutic gene alterations in rare tumors of Chinese population and suggest a treatment framework directed by molecular profiling.

Other genetic mutations also exhibited high prevalence in China. For example, cMET is aberrantly active in malignancy and involved in tumor growth and angiogenesis, and a cMET inhibitor (savolitinib) has been approved for the treatment of the 2–4% of non‐small cell lung cancers (NSCLC) that harbor *cMET* mutations (Li *et al*, 2022). Comparably, *cMET* mutations had a prevalence of 3.3% among the 3,453 Chinese rare tumor samples (Li *et al*, 2022).

Kirsten rat sarcoma (*KRAS*), a proto‐oncogene from the RAS/MAPK pathway, was considered undruggable for several decades due to the difficulty in direct targeting. Novel inhibitors targeting *KRAS* (G12C) such as sotorasib demonstrated promising results, and sotorasib has been approved by the FDA for NSCLC. A previous study in Chinese NSCLC patients reported a prevalence of 10.7% and 4.6% in overall *KRAS* mutation and G12C, respectively. We depicted the first *KRAS* mutation landscape of rare tumors in a large Chinese rare tumor population and observed an overall prevalence of 8.7% of *KRAS* mutation, concentrated on G12D, G12V, and G13D (Wang *et al*, 2022a). The G12C mutation was found in 0.6% of the population. However, a higher rate of G12C mutations was observed in sarcomatoid carcinoma of the lung (SARCL) and clear cell ovarian cancer (CCOV), with a prevalence of more than 5% (Wang *et al*, 2022a).

In recent decades, great progress has been made in immunotherapy for advanced cancer leading to a substantial survival benefit in multiple solid tumors, including gastric, liver, NSCLC, etc. We investigated the expression levels of programmed cell death ligand‐1 (PD‐L1) in 852 rare tumor patients, finding a prevalence of 47.8% positivity, which is higher than the prevalence found in common cancers with approved indications for immunotherapy (Wang *et al*, 2021). Both MSI and TMB are recognized predictive biomarkers for response to immune checkpoint inhibitors by the FDA in multiple solid tumors. These results support the potential benefit from immunotherapy in certain types of rare solid tumors.

Tumors harboring two or more druggable targets could potentially be responsive to a combination therapeutic strategy. For instance, *cMET* mutation was found to be highly consistent with PD‐L1 positivity (60.9%), suggesting a potential therapeutic interest in combining targeted therapy and immunotherapy in these patients.

## PLATFORM trial designs for rare tumors represents a successful model worldwide

For rare tumors, implementing a trial with enough participants for a specific disease is challenging, given that rare tumors are by nature uncommon in the general population. Platform trials including a range of rare tumor types with similar mutations may help circumvent this difficulty. Platform trials usually offer a pre‐screening genetic test, which helps directing the initial treatment for patients with *specific* mutations.

**Figure 2:**
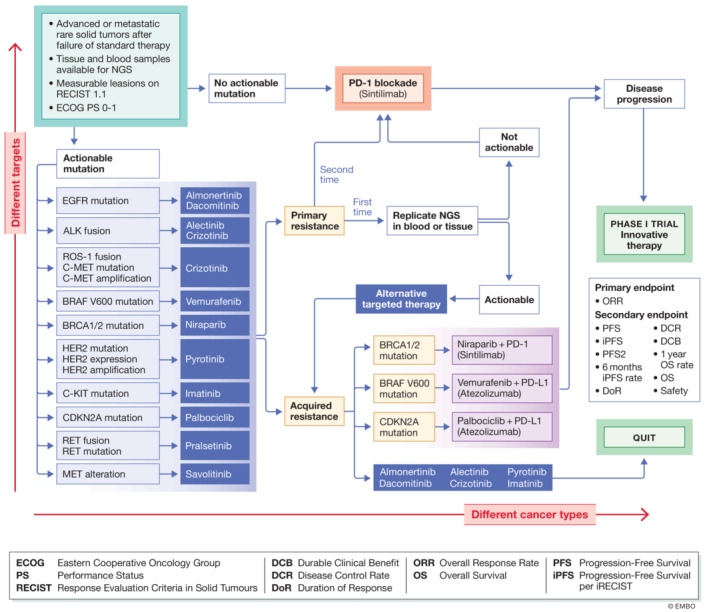
Schematic diagram of the PLATFORM study for rare solid tumors in China

The PLATFORM study, an open‐labeled, single arm, single‐center phase II trial in rare solid tumors is the first platform study specifically designed for rare solid tumors in a Chinese population (NCT04423185). The main purpose of the study is to evaluate the safety and efficacy of approved targeted therapies for patients with advanced rare solid tumor who have progressed on or lack standard treatment. The patients are recruited to the targeted therapy treatment groups based on the presence of specific tumor driver genes (Fig 2). The patients will receive immunotherapy (PD‐1 inhibitor monotherapy) if they have none of the listed druggable target mutations and are naïve to PD‐1/PD‐L1 inhibitor treatment. The PLATFORM study planned to recruit 1,557 participants, with 539 patients in the PD‐1 inhibitor treatment group. When exiting the PLATFORM study due to disease progression or drug intolerance, the patients will be introduced to other phase I trials of innovative therapies directed by the genetic data produced by this trial.

Other platform trials involving rare solid tumors include the National Cancer Institute–Molecular Analysis for Therapy Choice (NCI‐MATCH) trial in the United States, in which rare tumors account for more than 60% of the enrollment (Flaherty *et al*, 2020). The MASTER study (*Molecularly Aided Stratification for Tumor Eradication Research*) in Europe encompasses 75.5% of rare tumor patients. Evidence‐based management guided by genetic profiles resulted in significantly improved overall response and disease control rates compared with prior therapies in this trial. Japan also started the MASTER KEY Project, a biomarker‐driven platform trial for rare tumors, where significantly improved PFS was observed in the biomarker‐directed treatment group in the first interim analysis (Okuma *et al*, 2020).

## A blue ocean of drug investigation for rare tumors in China is awaiting

With a population of 1.4 billion and an annual cancer incidence of 4,820,000 cancer cases, rare tumors may not be all that rare in China. Thus, there is a huge unmet medical need for new effective treatments for patients with rare tumors. Based on recent literature, it is clear that there is a considerable therapeutic potential for the application of targeted treatments and immunotherapy in the Chinese rare tumor population. In the future, widespread use of gene panel testing will provide a more detailed description of the molecular characteristics for rare tumor subtypes and their relationship to treatment response. This will increase our understanding of the rare cancer genome and potentially help reduce morbidity and mortality of all rare tumors. However, it will necessitate to include rare or patient‐distinct variates in the clinical trials to gain better understanding and support clinical decision‐making and drug development.

On May 9, 2022, the National Medical Products Administration (NMPA) issued the Implementing Regulations of the Drug Administration Law (DAL) (Draft Amendments). The new DAL encourages the development and innovation of rare disease drugs. The review and approval of rare disease drugs that are urgently needed will be prioritized and the communication between the regulatory authorities and applicants will be strengthened. Furthermore, the drug marketing authorization holder of rare disease drugs may receive market exclusivity for up to 7 years. The reformed regulations exemplified by expedited approval programs in China have accelerated the drug development and increased the number of early phase trials (Wang *et al*, 2022b). The favorable regulatory and in‐market climate suggests that spring is coming for rare tumor drug development in China, and we should step up to tackle the critical clinical needs that are still unmet.

## Author contributions

**Shuhang Wang:** Conceptualization; funding acquisition; writing—review and editing. **Yale Jiang:** Investigation; visualization; writing—original draft; writing—review and editing. **Yuan Fang:** Investigation. **Ning Jiang:** Investigation; writing—review and editing. **Yue Yu:** Investigation. **Peiwen Ma:** Investigation. **Qiyu Tang:** Investigation. **Huilei Miao:** Investigation; writing—review and editing. **Dandan Cui:** Investigation. **Hong Fang:** Investigation. **Huiyao Huang:** Investigation. **Qi Fan:** Investigation. **Chao Sun:** Investigation. **Anqi Yu:** Investigation. **Shuangman Miao:** Investigation. **Jingting Du:** Investigation. **Jingxiao Zhu:** Investigation. **Yuning Wang:** Investigation. **Ning Li:** Conceptualization; funding acquisition; writing—review and editing.

## Disclosure and competing interests statement

The authors declare that they have no conflict of interest.
